# Assessment of water, sanitation and hygiene service availability in healthcare facilities in the greater Kampala metropolitan area, Uganda

**DOI:** 10.1186/s12889-020-09895-9

**Published:** 2020-11-23

**Authors:** Denis Kayiwa, Richard K. Mugambe, Jane Sembuche Mselle, John Bosco Isunju, John C. Ssempebwa, Solomon Tsebeni Wafula, Rawlance Ndejjo, Winnie K. Kansiime, Aisha Nalugya, Brenda Wagaba, Jude B. Zziwa, Constance Bwire, Esther Buregyeya, Martin Othieno Radooli, Ceaser Kimbugwe, Emily Namanya, Najib Lukooya Bateganya, Joanne A. McGriff, Yuke Wang, Tonny Ssekamatte, Habib Yakubu

**Affiliations:** 1 Programs Department, WaterAid Uganda, P.O. Box 11759, Kampala, Uganda; 2 Department of Disease Control and Environmental Health, School of Public Health, Kampala, College of Health Sciences, Makerere University, P.O Box 7072, Kampala, Uganda; 3 Department of Public Health, Kampala Capital City Authority, P.O Box 7010, Kampala, Uganda; 4 The Centre for Global Safe Water, Sanitation and Hygiene at Emory University, 1518 Clifton Rd. NE, Atlanta, GA 30322 USA

**Keywords:** Water, Sanitation and hygiene, Healthcare facilities, WASHCon, JMP, Uganda

## Abstract

**Background:**

Improved Water, Sanitation and Hygiene (WASH) in Healthcare facilities (HCFs) is of significant public health importance. It is associated with a reduction in the transmission of healthcare acquired infections (HAIs), increased trust and uptake of healthcare services, cost saving from infections averted, increased efficiency and improved staff morale. Despite these benefits, there is limited evidence on availability of WASH services in HCFs in the Greater Kampala Metropolitan Area (GKMA). This study assessed the availability and status of WASH services within HCFs in the GKMA in order to inform policy and WASH programming.

**Methods:**

A cross-sectional study was conducted in 60 HCFs. Availability of WASH services in the study HCFs was assessed using a validated WASH Conditions (WASHCon) tool comprising of structured interviews, HCF observations and microbial water quality analysis. Data were analysed using Stata 14 software and R software.

**Results:**

Overall, 84.5% (49/58) and 12.1% (7/58) of HCFs had limited and basic WASH service respectively. About 48.3% (28/58) had limited water service, 84.5% (49/58) had limited sanitation service, 50.0% (29/58) had limited environmental cleanliness service, 56.9% (33/58) had limited hand hygiene service, and 51.7% (30/58) had limited waste management service. About 94.4% of public HCFs had limited WASH service compared to only 68.2% of private not for profit facilities. More health centre IIIs, 92.5% and health centre IVs (85.7%) had limited WASH service compared to hospitals (54.5%).

**Conclusions:**

Our findings indicate that provision of water, sanitation, hand hygiene, environmental cleanliness, and health care waste management services within HCFs is largely hindered by structural and performance limitations. In spite of these limitations, it is evident that environmental cleanliness and treatment of infectious waste can be attained with better oversight and dedicated personnel. Attaining universal WASH coverage in HCFs will require deliberate and strategic investments across the different domains.

## Background

Water, Sanitation and Hygiene (WASH) in Healthcare facilities (HCFs) encompasses the provision of water, sanitation, health care waste management, hand hygiene and environmental cleanliness services [1]. Provision of WASH services in HCFs is fundamental for the provision of quality healthcare. Good WASH services in HCFs, especially in maternity and primary care settings have the potential to reduce healthcare acquired infections (HAIs), increase trust and uptake of healthcare services, increase efficiency and improve staff morale [1, 2]. In addition, improved WASH supports the core universal healthcare aspects of quality, equity, and dignity for all people [1].

Globally, WASH in HCFs remains a significant public health challenge. Global baseline estimates on WASH in HCFs indicate that 26% of HCFs lack access to an improved water source on the premises, 14% of HCFs have a limited water supply and 12% have no water supply at all [1]. Water service indicators are worse in low resourced countries where 45% of HCFs do not have access to basic water supply [1]. About 16% of HCFs globally also lack hand hygiene facilities at points of care, in addition to lacking soap and water at toilet facilities [1]. In terms of access to sanitation, over 21% of HCFs worldwide depend on unimproved toilets or have no toilets at all while in sub-Saharan Africa only 23% of HCFs have basic sanitation [1].

Inadequate WASH compromises safety and quality of healthcare services, and places a huge preventable risk to both health providers and clients [3]. Mothers and new-borns are at greater risk. Given the profound impact of WASH on patient dissatisfaction and quality of care [3], there is growing attention towards WASH in HCFs. WASH has for instance been recognised as key to the attainment of universal health coverage [1]. Besides, Sustainable Development Goal (SDG) 6 includes a target to achieve universal access to basic WASH for all including households, schools and HCFs, by 2030. However, even with just a decade to the evaluation of the attainment of the SDG 6, there is limited evidence on availability of WASH services in HCFs [1].

There is limited data on WASH in HCFs in urban Uganda, however, a study conducted in south western Uganda indicated critical gaps in the provision of WASH in HCFs in rural facilities [4]. Mulogo, Matte [4] reported that only 38% of the HCFs had toilets with hand washing facilities; with only 24% of the toilets having soap and water [4]. Similarly, Guo, Bowling [2] reported that less than 50% of rural HCFs in Uganda had access to improved water sources on premises, improved sanitation, and consistent access to water and soap for handwashing. Whereas Mulogo, Matte [4] and Guo, Bowling [2] give useful insights into WASH in HCFs in rural settings, their findings do not explicitly give a picture of availability of WASH services in a typically growing urban setting such as the Greater Kampala Metropolitan area (GKMA). Besides, their findings are not comparable to the Joint Monitoring Programme (JMP) WASH service ladders [1]. The JMP is an entity which was formed by the World Health Organization (WHO) and United Nations Children’s Fund (UNICEF) to produce internationally comparable statistics, and monitor progress towards attainment of SDG targets related to WASH. The JMP classifies water supply, sanitation, hygiene, waste management, and environmental cleanliness as basic, limited and no service [1].

In this study, we established and classified availability of WASH services in HCFs in the GKMA. This setting was chosen because of its representativeness of many growing cities in the Global South [5]. Findings from this study can be used to inform WASH programming and policy. Besides, data generated by this study can be used to track progress towards the attainment of national and international standards for WASH in HCFs.

## Methods

### Study setting and design

This cross sectional study utilised quantitative methods to collect data from selected public and private not for profit (PNFP) HCFs in the GKMA from January to March 2019. The GKMA includes the districts of Kampala, Wakiso and Mukono whose HCFs serve over 14% of Uganda’s population [6]. In this study, we considered HCFs at level III and above since these have a core mandate to deliver Maternal, New-born and Child Health (MNCH) services. In Uganda, the health care system is organised into a four-tier system (i.e., hospitals, health centres of levels IV, III and II) [7]. Level II health centres (HCs) have a catchment population of about 5000 people and only provide outpatient care and community outreach services. Level III HCs with a catchment population of about 20,000 people provide basic preventive, promotive, laboratory and curative services. They have limited inpatient capacity mainly maternity and general patient wards. Level IV HCs (catchment population 100,000) provide outpatient and inpatient services, maternity, children and adults’ wards, laboratory and blood transfusion services as well as an operating theatre. General hospitals (catchment population 500,000) provide preventive, promotive, curative, maternity, and inpatient health services and surgery, blood transfusion, laboratory, and medical imaging services.

### Sample size and sampling procedure

We sampled 60 out of 105 HCFs in the GKMA. In the sampling, we included all public and PNFP hospitals and HC IVs since these provide MNCH services to majority of the population in the GKMA. High volume PNFP hospitals and HC IVs were also purposively selected. We selected all the 8 PNFP hospitals, and 2 out of the 4 PNFP HC IVs. We purposively selected 28 out of 42 public, and 13 out of the 29 PNFP HC IIIs. HC IIIs with the largest catchment population were sampled.

### Data collection and measurement of study variables

Data collection was conducted using the validated WASH Conditions (WASHCon) tool on the Commcare mobile data collection platform. The tool, developed by the Centre for Global Safe Water, Sanitation, and Hygiene (CGSW) at Emory University has been used to evaluate WASH conditions within HCFs in low- and middle-income countries including Uganda [8–10]. The WASHCon tool relies on data collected through surveys, observational checklists and water quality testing. Data collection was done using mobile devices. The data was then uploaded into pre-programmed dashboards via a cellular or wireless internet network (not required during data collection). 

For this study, the outcome of the WASHCon tool was WASH service which was categorized as basic, limited or unimproved/no service similar to the JMP WASH service ladders [8]. Based on WASHCon indicators, WASH service is a composite variable generated from five variables (water, sanitation, environmental cleanliness, hand hygiene and waste management services). In order to establish the water service, data was collected on source and accessibility, quantity and quality of water. Sanitation service was assessed by collecting data on accessibility to toilet facilities, number of toilets and existence of the infrastructure, while for hand hygiene services data was collected on availability of hand hygiene facilities and availability of associated supplies. Assessment of environmental cleanliness service was based on availability of cleaning supplies, cleaning practices and frequency, and facility hygiene. In order to establish the availability of waste management service, data were collected on segregation, treatment and disposal of healthcare waste.

Using the WASHCon dashboard, evaluation scores were calculated on a scale of 1–3 for each of the WASH domains, as well as an overall score that is an average of all the domains. The scores were determined based on the responses to the survey questions, observation checklists, and water quality testing results (Additional file 1). These scores were further categorized into basic, limited or unimproved/ no service. HCFs that scored between 2.8 to 3.0 were classified as basic, and were considered to meet the minimum WASH in HCF requirements or were on track to meet them; HCFs that scored between 1.9 to 2.7 were classified as limited, and were considered to have made some progress towards meeting minimum requirements for WASH in HCFs but were not on track to meet them; while HCFs that scored between 1.0 to 1.8 were classified as having no service or unimproved (Additional file 1). Such facilities were considered to have made little or no progress towards achieving the minimum requirements for WASH in HCFs [8]. The independent variables included ownership (public vs. PNFP) and level of facility (HC III, HC IV and Hospital).

### Quality control

Prior to data collection, study enumerators received training on the use of the WASHCon tool, quality control and research ethics. The observations and interviews were conducted by trained enumerators who had a minimum of a Bachelor’s degree in Environmental Health Science; Nursing; or Social Sciences. All the study enumerators were supervised to ensure quality control.

### Microbial water quality analysis

In order to determine the availability of water services in HCFs, observations were done to establish the type of water source and availability of water, and this was followed by collection of duplicate water samples for microbial analysis. Water samples were collected from maternity wards, which were prioritised due to an elevated risk of transmission of HAIs compared to other patient care areas [11]. Water samples were collected using Whirl-Pak bags of 100 mls (with sodium thiosulfate to halt chlorine action in chlorinated supplies) and stored on ice until laboratory analysis. All samples were analysed within 4 hours from the time of collection. Water was tested for faecal coliform, i.e. *E. coli* using the membrane filtration method [12]. Chromocult agar was used for culturing E-*Coli* at 37 °C for 24 h. Colonies of *E-coli* (i.e. dark blue to violet in colour) were counted and results recorded per 100 ml of sample.

### Data management and analysis

The data obtained using the WASHCon Commcare app, preinstalled on a mobile device were uploaded onto a server managed by Makerere University School of Public Health and Emory University CGSW. Forms were synchronized daily by each enumerator. The investigators had access to preliminary results through a pre-programmed dashboard.

Analysis was performed using Stata version 14 (StataCorp, Texas) and R 3.5.2. Descriptive statistics such as frequencies and proportions were used to summarize quantitative categorical data. Continuous data were expressed as means and standard deviations. Classification of WASH service and its five domains into basic, limited and unimproved/no service was guided by the scoring tool shown in additional file 1.

## Results

### Characteristics of study health facilities

Among the 60 HCFs, 31.7% (19/60) were from Kampala capital city; and about 70.0% (42/60) of HCFs were HC IIIs. About 65.0% (39/60) were public HCFs. The average number of patients seen at these HCFs in a month was 3718. The number of patients seen at the HCFs per month ranged from 6 to 50,200 (Table 1).

**Table 1:** Characteristics of study healthcare facilities

Description.	Characteristic	Frequency (***N*** = 60)	Percentage (%)
District	Kampala	19	31.7
	Mukono	14	23.3
	Wakiso	27	45.0
Level of HCF	Health centre III	42	70.0
	Health Centre IV	7	11.7
	Hospital	11	18.3
Ownership	Public	39	65.0
	PNFP	21	35.0

### Water service

Most HCFs, 70.7% (41/60), depended on piped water supply. In 93.1% (54/58) of the HCFs, the primary source of water was on facility premises. At the time of the survey, water from the main source was available at 93.3% (56/60) of the HCFs. In about 58.3% (35/60) of the HCFs, water was piped into the wards. Three quarters 75.9% (44/60) of the HCFs had previous instances of water discontinuity. Overall, 87.9% (51/58) of the HCFs had water that met the WHO microbial drinking water quality guidelines of 0 coliform forming units per 100 ml of water (Table 2).

**Table 2:** Availability of water service in healthcare facilities in the GKMA, Uganda

Description	Characteristic	Frequency (***n*** = 60)	Percentage (%)
Main water source (*n* = 58)^a^	Borehole	1	1.7
	Protected dug well	2	3.4
	Piped supply from outside the HCF	41	70.7
	Rainwater	13	22.4
	Tanker truck	1	1.7
Location of main water source (n = 58)^a^	Off premises, within 500 m	3	5.2
	Off premises, further than 500 m	1	1.7
	On premises	54	93.1
Instances of discontinuity (when water from the main water source is unavailable?) (n = 58)^a^	Yes	44	75.9
	No	14	24.1
Frequency of discontinuity in water supply (*n* = 58)^a^	For part of the day, frequently	9	15.5
	For part of the day, rarely	15	25.9
	For part of the year (seasonal problem frequently)	8	13.8
	For part of the year (seasonal problem rarely)	11	19.0
	Water is always available	14	24.1
	Do not know	1	1.7
HCF at times faces a severe water shortage (*n* = 44)^a^	Yes	14	31.8
	No	28	63.7
	Don’t know	2	4.5
HCF has an alternative water source	Yes, and the alternative source is improved	36	60.0
	Yes, but the alternative source is unimproved	3	5.0
	No alternative water source	17	28.3
	Have alternative source but water is unavailable	3	5.0
	Do not know	1	1.7
Water accessible to all users at all times at HCF	Yes	55	94.8
	No, patients/caregivers do not have access at times	2	3.4
	No, both staff and patients/caregivers do not have access at times	1	1.8
Water is available from the main water source at the time of the survey	Yes	56	93.3
	No	4	6.7
Is water piped into the wards	Yes	35	58.3
	Yes, but currently unavailable	3	5.0
	No	22	36.7
Water at HCF meets the WHO microbial water quality standards (n = 58)^a^	< 50% of all samples met	6	10.3
	50–89% of all samples met	1	1.7
	90–100% of all samples met	51	87.9

Note: ^a^sample size less than 60 due to missing data

### Sanitation service

One HCF had no sanitation facility. About, 25.9% (15/58) of the HCFs used onsite sanitation systems. More than half, 68.3% (41/59) of the HCFs did not provide for menstrual hygiene needs. Only 20.0% (12/58) of the HCFs had improved toilets that meet the needs of people with reduced mobility (Table 3).

**Table 3:** Sanitation service in healthcare facilities in the GKMA, Uganda

Description	Characteristic	Frequency (n = 60)	Percentage (%)
Type of toilet facilities available at HCF	Flush	23	38.3
	Pour flush	4	6.7
	Pit latrine with a slab	7	11.7
	Ventilated Improved Pit latrine	20	33.3
	Pit latrine without a slab	5	8.3
	No toilet facility	1	1.7
Method of excreta disposal at the HCF (n = 58)^a^	Sewerage system	14	24.1
	Septic tank	15	25.9
	Pit or chamber	28	48.3
	No toilet facility	1	1.7
Main users of toilet blocks at HCF	Both staff and patients/ caregiver	16	26.7
	Both staff and patients/ caregiver, but separated	15	25.0
	Patients/ caregivers only	28	46.7
	Staff only	1	1.7
Presence of flies in improved toilets	Present in all toilets	5	8.3
	Absent in all toilets	41	68.3
	Present in some toilets	13	21.7
	No toilet facility	1	1.7
Presence of unpleasant smell (of urine or faeces) at the toile/latrine block	Yes	27	45.0
	No	32	53.3
	No toilet facility	1	1.7
Visible cleanliness of HCF toilet blocks	Yes	45	75.0
	No	14	23.3
	No toilet facility	1	1.7
Adequacy of lighting at the toilet blocks, including at night	Yes	32	53.3
	No	27	45.0
	No toilet facility	1	1.7
Provision for menstrual hygiene needs (*n* = 59)^a^	Yes	18	30.5
	No	41	69.5
Presence of lockable doors	Present on all toilets	56	93.3
	Present on some toilets	1	1.7
	No toilet facility	1	1.7
Presence of an improved toilet that meets the needs of people with reduced mobility (n = 58)^a^	Present	12	20.0
	Absent	46	76.7
	Did not observe	1	1.7
	No toilet facility	1	1.7
Gender-based separation of toilet blocks	Both males and females, unseparated	9	15.0
	Both males and females, but separated	43	71.7
	Females only	8	13.3

Note: ^a^sample size less than 60 due to missing data

### Hand hygiene service

Over 41.6 (25/60) of the HCFs did not have a fully functional hand hygiene facility (HHF) in patient care areas; and only 56.6% (34/60) had functional HHF with soap and water within five metres of the toilet block (Table 4).

**Table 4:** Hand Hygiene service in healthcare facilities in the GKMA

Description	Characteristic	Frequency (n = 60)	Percentage (%)
Status of hand hygiene facilities in the patient care areas	No supplies available	6	10.0
	Water only	17	28.3
	Water and sanitiser available	1	1.7
	Water and soap available	35	58.3
	Water, soap and sanitiser available	1	1.7
HCF has functioning hand hygiene facilities within five meters of the toilet block	Hand hygiene facilities available but non- functional	2	3.3
	Did not observe	1	1.7
	No toilet facility	1	1.7
	Hand hygiene facilities not available	6	10.0
	Hand hygiene facilities available with water only	24	40.0
	Hand hygiene facilities available with both water and soap	26	43.3

### Environmental cleanliness service

Open defecation was practiced at one of the HCFs. Majority of the HCFs, 81.7% (49/60) had proper containment of faeces from babies. Most HCFs had patient care areas with visibly clean wards 86.7% (52/60), and 88.3% (53/60) had clean floors. However, 3.3% (2/60) had uncleaned spills from bodily fluids. About 20.0% (12/60) of the HCFs had uncontained solid waste. About 79.3% (46/58) and 6.9% (4/58) of the HCFs reported cleaning beds, mattresses, pillowcases or mats always and sometimes respectively between patients but in 12.1% (7/58) beddings were not provided. (Table 5).

**Table 5:** Environmental cleanliness service in healthcare facilities in the GKMA, Uganda

Description	Characteristic	Frequency (n = 60)	Percentage (%)
Open defecation practiced (visible faecal matter at the HCF)	No	57	95.0
	Yes	1	1.7
	Did not observe (No access)	2	3.3
Containment of babies’ faeces	Well contained	49	81.7
	Not well contained	10	16.7
	Didn’t observe	1	1.7
HCF has uncontained solid waste	Yes	12	20.0
	No	48	80.0
HCFs had clean floors	Yes	53	88.3
	No	7	11.7
Beds, mattresses, pillows and/or mats cleaned between patients	Yes, always	46	79.3
	Yes, sometimes	4	6.9
	No inpatient	1	1.7
	Beddings not provided	7	12.1
HCFs have unclean bodily spills	Yes	2	3.3
	No	58	96.7

### Waste management service

Majority 63.2% (36/57) of the HCFs had fenced and protected areas for the storage of healthcare waste (HCW) awaiting disposal or removal. More than half, 58.6% (34/58) did not treat infectious waste before disposal and 27.6% (16/58) of the HCFs disposed infectious waste offsite. More than half, 58.6% (34/58) of HCFs did not treat sharps waste most of the time while 37.9% (22/58) disposed these sharps offsite. Waste segregation into at least three labelled bins (for sharps waste, infectious waste and non-infectious general waste) was done in 85.0% (51/60) of the HCFs (Table 6).

**Table 6:** Waste management service in healthcare facilities in GKMA

Description	Characteristic	Frequency, n	Percentage (%)
Availability of protected areas for the storage of HCW awaiting disposal or removal (*n* = 57) ^a^	Yes	36	63.2
	Sometimes	2	3.5
	No	19	33.3
Commonest means of treating infectious waste (n = 58) ^a^	Autoclave	4	6.9
	Chemical disinfection with hypochlorite	12	20.7
	Not treated	34	58.6
	Other	8	13.8
Commonest method of disposal of infectious waste disposed? (*n* = 58) ^a^	Incinerate (brick incinerator)	12	20.7
	Incinerate (two chamber, 850–100 °C)	4	6.9
	Burn in protected pit	5	8.6
	Burry in a lined, protected pit	2	3.4
	Collect for medical waste disposal offsite	16	27.6
	Collected for general waste disposal offsite	4	6.9
	Open burning	14	24.1
	Don’t know	1	1.7
Main treatment method for sharps (n = 58) ^a^	Autoclave	4	6.9
	Chemical disinfection with hypochlorite	6	10.3
	Not treated	34	58.6
	Other	12	20.7
	Don’t know	2	3.4
Main disposal method for sharps (n = 58) ^a^	Incinerate (brick incinerator)	11	19.0
	Incinerate (two chamber, 850–100 °C)	3	5.2
	Burn in protected pit	6	10.3
	Collected for medical waste disposal offsite	22	37.9
	Collected for general waste disposal offsite	4	6.9
	Open burning	11	19.0
	Don’t know	1	1.7
Waste safely segregated into at least three labelled bins (for sharps, infectious waste and non-infectious waste)	Yes	51	85.0
	Bins present but waste is not segregated	7	11.7
	No	2	3.3

Note: ^a^sample size less than 60 due to missing data

### WASH service based on JMP service ladders

The average scores (SD) for the WASH service domains were: 2.6 ± 0.3 for water service; 2.1 ± 0.2 for sanitation service; 2.4 ± 0.5 for environmental cleanliness service; 2.4 ± 0.4 for hand hygiene service; and 2.5 ± 0.4 for waste management service. The overall average score for WASH service was 2.4 ± 0.2. Overall, only 12.1% (7/58) of HCFs were found to have basic WASH service; 48.3% (28/58) had a limited water service, 84.5% (49/58) had a limited sanitation service, 50.0% (29/58) had limited environmental cleanliness service, 56.9% (33/58) had a limited hand hygiene service and 51.7% (30/58) had a limited waste management service.

### Availability of WASH services based on ownership and level of health facility

Overall, 94.4% of public and 68.2% of PNFP HCFs had a limited WASH service. About 61.1% of public HCFs had a limited water service, 16.7% had an unimproved/no sanitation service, 8.3% had an unimproved/no environmental cleanliness service, 63.9% had a limited hand hygiene service, and 2.8% had an unimproved/no waste management service. Regarding level of the HCF, 60.0% of HC IIIs had a limited water service, 14.3% of HC IVs had an unimproved/no sanitation service, 28.6% of HC IVs had an unimproved/no environmental cleanliness service, and 10% of the HC IIIs had an unimproved/no hand hygiene service. None of the hospitals and HC IVs had an unimproved/no waste management service (Table 7).

**Table 7:** WASH service ladders based on healthcare facility characteristics

Score (Range)	Characteristic	Overall frequency (n) (%)	Ownership		Level of healthcare facility		
			Public	PNFP	Hospital	HC IV	HC III
**Overall WASH service** (Min score = 1.5, 3.0)	Basic	7 (12.1)	2 (5.6)	5 (22.7)	5 (45.5)	1 (14.3)	1 (2.5)
	Limited	49 (84.5)	34 (94.4)	15 (68.2)	6 (54.5)	6 (85.7)	37 (92.5)
	Unimproved/No service	2 (3.4)	0 (0)	2 (9.1)	0 (0)	0 (0)	2 (5.0)
**Water service** (1.6, 3.0)	Basic	27 (46.6)	13 (36.1)	14 (63.6)	11 (100)	3 (42.9)	13 (32.5)
	Limited	28 (48.3)	22 (61.1)	6 (27.3)	0 (0)	4 (57.1)	24 (60.0)
	Unimproved/No service	3 (5.2)	1 (2.8)	2 (9.1)	0 (0)	0 (0)	3 (7.5)
**Sanitation service** (1.5, 3.0)	Basic	2 (3.4)	0 (0)	2 (9.1)	0 (0)	1 (14.3)	1 (2.5)
	Limited	49 (84.5)	30 (83.3)	19 (86.4)	11 (100)	5 (71.4)	33 (82.5)
	Unimproved/No service	7 (12.1)	6 (16.7)	1 (4.5)	0 (0)	1 (14.3)	6 (15.0)
**Environmental cleanliness service** (1.0, 3.0)	Basic	23 (39.7)	13 (36.1)	10 (50.0)	6 (54.5)	1 (14.3)	16 (40.0)
	Limited	29 (50.0)	20 (55.6)	[9] 45.5	5 (55.4)	4 (57.1)	23 (57.5)
	Unimproved/No service	6 (10.3)	3 (8.3)	2 (9.1)	0 (0)	2 (28.6)	1 (2.5)
**Hand hygiene service** (1.4, 3.0)	Basic	21 (36.2)	10 (27.8)	11 (50.0)	6 (55.5)	2 (28.6)	13 (32.5)
	Limited	33 (56.9)	23 (63.9)	10 (45.5)	5 (45.5)	5 (71.4)	23 (57.5)
	Unimproved/No service	4 (6.9)	3 (8.3)	1 (4.5)	0 (0)	0 (0)	4 (10.0)
**Waste management service** (1.5, 3.0)	Basic	27 (46.6)	16 (44.4)	11 (50.0)	9 (81.8)	2 (28.6)	16 (40.0)
	Limited	30 (51.7)	19 (52.8)	11 (50.0)	2 (18.2)	5 (71.4)	23 (57.5)
	Unimproved/No service	1 (1.7)	1 (2.8)	0 (0)	0 (0)	0 (0)	1 (2.5)

Note: *PNFP* Private not for profit

## Discussion

This study assessed the availability of WASH services in HCFs in an urban setting in Uganda. Though existing literature on WASH in HCFs in low resource settings indicates limited access to improved water sources, this study found out that almost all the HCFs in the GKMA had access to an improved water source, however, access rates remain below the WHO target of 100% coverage by 2030 [13]. The high access to improved water sources in our study could be attributed to deliberate efforts by the government and line ministries to invest in improved access to safe water in urban settings. For Uganda’s case, our findings are not different from those of rural settings as reported by Mulogo, Matte [4]. While almost all HCFs had an improved water source, a significant proportion did not have an alternative water source. Lack of an alternative source may compromise access during times of seasonal scarcity and breakdown of water facilities.

More than a quarter of the HCFs in the study area reported experiencing intermittent water supply, and often suffered severe water shortage. These results are similar to those of a study conducted in Rwanda which indicated seasonal water shortages in HCFs [14]. Besides seasonal shortages, intermittent water supply in HCFs in Uganda could also be related to failure of HCFs to pay water bills. In addition, the national utility which is mandated to supply water in the urban areas often fails to meet the water demand [15, 16]. This poses a serious challenge in urban settings where the population and the number of clients seeking care from HCFs are large. Therefore, intermittent water supplies could provide an environment for opportunistic infections especially among immunocompromised patients such as the new-borns and mothers.

In a tenth of the HCFs, 50% of the water samples met the recommended WHO microbial water quality standards of 0 CFU per 100 mL. Water samples that did not meet the WHO microbial water quality standards were mainly from HCFs whose main water source was not piped. Unlike other water sources, piped water in the GKMA is treated with chlorine, a highly efficient disinfectant which reduces the risk of faecal contamination. Therefore, the presence of *E.coli* in drinking water in a tenth of the HCFs suggests faecal pollution and presents a serious potential hazard in those HCFs [17]. Our finding is similar to a previous study by Huttinger, Dreibelbis [14] in which over 25% of water samples in selected rural HCFs in Rwanda did not meet the WHO standards of microbial water quality. The low microbial quality of water in urban HCFs in the GKMA could be attributed to contamination resulting from pipe leakages, lack of clean storage reservoirs such as water tanks and poor environmental sanitation surrounding the water sources [18].

Our study indicates that almost all HCFs in the study area had a sanitation facility, which was either jointly used by staff and patients or separated for either categories. Although stances for both patients and staff could be on the same latrine block, it is a common practice for the stances used by healthcare staff to be kept under lock and key for purposes of maintaining them clean. The fact that most HCFs had adequate sanitation facilities enables provision of quality healthcare [1]. Our findings corroborate those of a study in Jordan where all HCFs had sufficient toilets [19]. Despite availability of sanitation facilities in most of the HCFs in our study, about 71.7% of these facilities were not gender sensitive. The low gender sensitivity in toilet design may affect proper usability of these facilities due to issues of privacy and comfort. Huttinger, Dreibelbis [14] in their study also highlighted lack of gender sensitive sanitation facilities in HCFs. Unhygienic conditions of visible flies, unpleasant smells and visibly unclean toilets were common. The unpleasant smells that characterise sanitation facilities in the GKMA could be related to inadequate funding for WASH services, and consequently poor cleaning routines. This study also brings to light a lack of menstrual hygiene facilities in HCFs. Though rarely studied, a lack of menstrual hygiene facilities could result into patient dissatisfaction with health care services [1]. Therefore, provision of these menstrual hygiene facilities would improve usability of sanitary facilities. From our study, we reveal that a significant proportion of sanitation facilities in healthcare settings lack adequate lighting, most especially at night. This is likely to affect usability of the facilities and may result into indiscriminate excreta disposal. Adequate lighting in sanitation facilities should be ensured since lighting increases feelings of security and safety for users and encourages their optimal use [20, 21]. It is worth noting that 68.3% of the sanitation facilities in the surveyed HCFs did not have flies. This could be attributed to the fact that a significant proportion of HCFs had improved sanitation facilities.

Hand hygiene remains a significant challenge in HCFs. This study revealed that only 58% of the HCFs had at least one functional hand hygiene facility with both water and soap in patient care areas. This low proportion of functional hand hygiene facilities indicates potential for elevated risk of for transmission of HAIs at points of care across HCFs. Our findings differ from a study conducted by Mulogo, Matte [4] that revealed that only 24% of the HCFs in south-western Uganda had water and soap present at the hand washing stations. The disparity in these findings could be related to the fact that our study was conducted in an urban area with considerably more WASH investments as compared to Mulogo’s study which was conducted in predominantly rural HCFs. Lack of functional hand hygiene facilities in HCFs is likely to compromise infection prevention and control efforts for highly infectious diseases such as Ebola and COVID-19. Furthermore, less than half of the HCFs had a functional hand hygiene facility with water and soap within 5 m of the toilet block, similar to a study by Guo, Bowling [2] which showed that only a small proportion of HCFs in Uganda have water and soap available for hand washing near the toilets. The low proportion of hygiene facilities with water and soap may be attributed to limited funds to put up and sustain functioning hand hygiene facilities that meet the basic requirements at the HCF. This indicates a need for more financial investments but also improve attitudes among both health care in charges and administrators.

It was noted in this study that most HCFs segregated waste safely into separate bins, contrary to what has been reported in previous studies about the absence of proper waste segregation practices at the point of generation in HCFs [22–24]. More than half of the HCFs did not treat infectious waste and sharps most of the time. These findings concur with those of an Ethiopian study, where there was no pre-treatment of infectious wastes by the HCFs [25]. This implies that health workers, waste handlers and the public could be at risk of infections from the waste. Nonetheless, majority the HCFs had protected areas for the storage of HCW awaiting disposal. Protected waste storage areas can minimize risks of potential injuries and infection, particularly among the public and stray animals venturing to the waste sites are deterred.

In comparison with the JMP indicators, this study revealed that majority of the HCFs had a limited WASH service. This indicates gaps in WASH in HCFs and the need for more investments for attainment of optimal WASH services. Limited investment in WASH in HCFs could partly explain this. To put this into context, any improvements in WASH in HCFs in Uganda are dependent on the availability of the already meagre primary healthcare funds [26]. Therefore, with limited funds and regular financial needs, HCFs may have to make a trade-off between financing WASH services and sustaining other HCF operations such as paying non-wage staff and paying off other utility bills such as electricity.

From this study, there is some evidence that the provision of WASH services differs across the ownership and level of HCFs. More PNFP HCFs had better WASH services compared to the public HCFs. It has been assumed that private HCFs at the same level as public HCFs generally have better service standards [4]. Unlike public HCFs where services are free, PNFP HCFs in the study area provide healthcare services at a cost. Therefore, the funds generated from the provision of these healthcare services may be used to increase the budget for WASH. In addition, PNFP HCFs are interested in attracting more clientele so they may have more deliberate efforts to improve WASH so as to attract more clients and ensure patient satisfaction.

A higher proportion of hospitals in the study area had an overall basic WASH service based on JMP service ladders compared to the lower level HCFs. This could be attributed to the fact that hospitals in Uganda are given more primary healthcare funds to support improvements in WASH, given that hospitals have relatively large population catchments and offer a wider range of MNCH services. In addition, hospitals are often accorded more attention due to a higher patient load and a higher risk of transmission of hospital acquired infections compared to the lower level HCFs. The higher patient load in hospitals could also trigger more investments in WASH services due to the fear of transmission of HAIs, thus a higher proportion having an overall basic WASH supply [27].

## Conclusions

Overall, majority of the HCFs had access to improved water sources and sanitation facilities but few had functional hand hygiene facilities. However, based on the JMP service ladders, majority of the HCFs had a limited WASH service. The WASH service significantly differed across the different levels and ownership of HCF. Our findings demonstrate structure and performance limitations in provision of WASH services in HCFs, and indicate the need for deliberate and strategic investments in healthcare WASH services, especially in terms of finances, infrastructure and policies. The present study also reveals that environmental cleanliness and treatment of infectious waste can be achieved in the absence of infrastructure improvements, if there is better oversight and personnel to do it. Improvements in WASH conditions will not only minimize the risk of transmission of hospital acquired infections but also may cut on associated costs. We therefore suggest improvements in WASH conditions in HCFs to improve healthcare seeking among patients.

## Supplementary Information

**Additional file 1.** Definitions of WASH status indicators (domains)

## Data Availability

The datasets analysed during the current study are available from the corresponding author on reasonable request.

## Abbreviations

CGSW: Centre for Global Safe Water, Sanitation, and Hygiene
GKMA: Greater Kampala Metropolitan Area
HAIs: Healthcare Acquired Infections
HC: Health Centre
HCF: HealthCare Facility
JMP: Joint Monitoring Programme
PNFP: Private Not for Profit
SDG: Sustainable Development Goal
WASH: Water, Sanitation and Hygiene
WASHCon: WASH Conditions
