# Supplementary material for: Assessment of water, sanitation and hygiene service availability in healthcare facilities in the greater Kampala metropolitan area, Uganda
Source: BMC Public Health. 2020 Nov 23;20:1767. doi: 10.1186/s12889-020-09895-9 (PMC7682765; doi:10.1186/s12889-020-09895-9)
Supplement: Supplementary file 1 — Additional file 1. Definitions of WASH status indicators (domains) [file 12889_2020_9895_MOESM1_ESM.docx]

**Additional file 1 Definitions of WASH status indicators (domains)**

| Domains (Indicators) | **Service level** | | |
| --- | --- | --- | --- |
|  | Basic  **(Scores 2.8-3.0)** | Limited  **(Scores 1.9-2.7)** | Unimproved / No Service  **(Scores 1.0-1.8)** |
| **Water services** | Water from an improved source is available on premises | Water from an improved source is available off premises; or an improved source is onsite, but no water is available | Unprotected dug well or spring, surface water, or no water source |
| **Sanitation services** | Improved facilities are usable, separated for patients and staff, separated for women, provide menstrual hygiene facilities, and meet the needs of people with limited mobility | Improved sanitation facilities are present but are not usable or do not meet the needs of specific groups (staff, women, people with limited mobility) | Pit latrines without a slab or platform, hanging latrines, or no toilets or latrines at the facility |
| **Hand hygiene services** | Hand hygiene materials, either a basin with water and soap or alcohol hand rub, are available at points of care and toilet | Hand hygiene station at either point of care or toilets, but not both | Hand hygiene stations are absent, or present but with no soap or water |
| **Waste management services** | Waste is safely segregated into at least 3 bins in the consultation area, and sharps and infectious waste are safely treated and disposed of | Waste is segregated but not disposed of safely, or bins are in place but not used effectively | Waste is not segregated or safely treated and disposed of |

*Adapted from the JMP service ladders for monitoring WASH in HCF in the SDGs*
